# Non-native gobies facilitate the transmission of *Bucephalus polymorphus* (Trematoda)

**DOI:** 10.1186/s13071-015-0999-7

**Published:** 2015-07-19

**Authors:** Markéta Ondračková, Iveta Hudcová, Martina Dávidová, Zdeněk Adámek, Martin Kašný, Pavel Jurajda

**Affiliations:** Institute of Vertebrate Biology, v.v.i., Academy of Sciences of the Czech Republic, Květná 8, 603 65 Brno, Czech Republic; Department of Botany and Zoology, Faculty of Science, Masaryk University, Kotlářská 2, 611 37 Brno, Czech Republic; Department of Parasitology, Faculty of Science, Charles University in Prague, Viničná 7, 128 44 Prague, Czech Republic

**Keywords:** Trematode, *Bucephalus polymorphus*, Complex life cycle, Non-native species, Infectivity, Intermediate host, Goby

## Abstract

**Background:**

Introduced species can modify local host-parasite dynamics by amplifying parasite infection which can ‘spill-back’ to the native fauna, whether they are competent hosts for local parasites, or by acting as parasite sinks with ‘dilution’ of infection decreasing the parasite burden of native hosts. Recently infection by the trematode *Bucephalus polymorphus* has increased in several European rivers, being attributed to the introduction of intermediate host species from the Ponto-Caspian region. Using a combination of field and experimental data, we evaluated the competence of non-native and native fish as intermediate hosts for *B. polymorphus* and its role for parasite development in a definitive host.

**Methods:**

The density of 0+ juvenile fish (the second intermediate hosts for *B. polymorphus*) was measured in the River Morava, Czech Republic and fish were screened for natural metacercariae infection. The stomach contents of predatory fish that are definitive hosts of *B. polymorphus* were examined to assess the importance of non-native gobies for parasite transmission. In semi-natural conditions, parasite establishment, initial survival, and maturity rates in experimentally infected definitive hosts pikeperch *Sander lucioperca* were measured in flukes recovered from native white bream *Abramis bjoerkna* and non-native tubenose goby *Proterorhinus semilunaris* and round goby *Neogobius melanostomus*. Adult fluke size and egg production was also measured to evaluate the potential effect of intermediate host species on parasite fitness.

**Results:**

We detected high natural infection parameters of *B. polymorphus* in native cyprinids and non-native gobies compared to data from the period prior to goby establishment. Both fish groups are consumed by predatory fish and represent a major component of the littoral fish community. Parasite establishment and adult size in definitive hosts was equivalent among the second intermediate host species, despite a lower size of metacercariae recovered from round gobies. However, development in the definitive host of flukes recovered from gobies was reduced, showing higher mortality, delayed maturity and lower egg production, in comparison with parasites from native hosts.

**Conclusions:**

Substantial ‘spill-back’ of *B. polymorphus* due to higher transmission rates after establishment of non-native gobies was partially buffered by decreased fitness of *B. polymorphus* that underwent development in gobies.

## Background

The role that parasites can play in biological invasions has currently received increased interest. Through their effects on host fitness, population growth, and by modifying interactions between native and introduced species (including competition and predation), parasites may play a key role in determining the success of an invasion and its impact on the recipient community [1, 2]. In a new environment, introduced species often tend to accumulate generalist parasites from the local fauna over time [3], although their abundance and prevalence is usually lower compared to native hosts [4].

When acquiring local parasites, non-native hosts may serve as alternative hosts from which the parasite may ‘spill-back’ to the native fauna [5]. When a new species represents a competent host in which the parasite can develop normally, the total number of parasites to which native hosts are exposed is amplified and may potentially lead to an emerging disease [6]. On the other hand, if parasites cannot develop in the new host species but infect it anyway, then it may serve as a sink for the parasite population, leading to reduction in infection levels in native hosts via a ‘dilution’ effect [7]. In addition to directly amplifying infection of native parasites by acting as a definitive host, non-native species may also potentially increase infection by native parasites in native hosts by fulfilling other roles in the parasite life cycle, such as that of intermediate host or vector (termed trophically mediated ‘spill-back’). Such ‘spill-back’ then may potentially be driven by differences in the behaviour or ecology of non-native species compared to native intermediate hosts or vectors [5].

Within the life cycles of heteroxenous parasites, fish frequently serve as an intermediate or paratenic hosts. In trematodes, the dominant internal parasites of teleost fishes [8], the host specificity at the level of fish intermediate host is often low. Metacercarial stages utilizing fish as their second intermediate host comprise a significant component of parasite communities in a wide range of fish species, especially those of relatively small size that can readily be preyed upon by parasite definitive hosts (e.g. [9, 10]). Dominance of non-specific larval parasites in parasite communities of non-native goby populations has been frequently observed (e.g. [11–14]), highlighting the importance of non-native host species for parasite dynamics in new habitats. In species achieving high densities and spreading rapidly in novel areas, acquisition of high numbers of larval parasite species and individuals may lead to an increase in parasite abundance and dispersal if the new host is both competent for the parasite and utilized as a prey by the parasite definitive host. In the case that a non-native host is avoided by predators, the life cycle is not completed and the new host would serve as a dead-end host despite its susceptibility to the parasite [15].

In this study, we investigated the role of recently expanding Ponto-Caspian goby fishes (western tubenose goby *Proterorhinus semilunaris* and round goby *Neogobius melanostomus*, Gobiidae) in the River Morava, Czech Republic (a tributary of the Danube) [16] as intermediate hosts for metacercariae of *Bucephalus polymorphus* (Trematoda, Bucephalidae). These Ponto-Caspian gobies are invasive species that were introduced in ballast water to various river systems in Europe and North America during recent decades [4, 16]. *B. polymorphus* is a trematode parasite infecting the intestines of predatory fish (e.g. Percidae, Esocidae, etc.). Within its life cycle, unionid and dreissenid bivalves serve as the first intermediate host; a wide range of fish species serve as the second intermediate host; and piscivorous fish serve as definitive hosts. The parasite exhibits low specificity for the second intermediate host, having been reported from a range of freshwater, brackish and marine fishes [17]. The occurrence of *B. polymorphus* in the River Morava basin was first recorded in the 1950s, from both intermediate (mainly cyprinid) and definitive (mainly percid and esocid) fish hosts (summarized in [18]). High susceptibility of Ponto-Caspian gobies to *B. polymorphus* in their non-native range has recently been reported from the middle Danube [19] and River Vistula [20]. Although this parasite is known to use some goby species as intermediate hosts in the Black Sea basin in estuaries [21], infection is rare [22]. The occurrence of *B. polymorphus* in gobies, however, has not been documented from the freshwater section of the lower Danube [23–27], the original area of fish established in the middle Danube and its tributaries, such as the River Morava.

To examine whether infection by *B. polymorphus* in non-native gobies may lead to parasite-mediated ‘spill-back’, we combined field data and experimental infection of potential definitive hosts. In the field, we estimated the density of native and non-native fish (intermediate hosts) that potentially serve as prey for predatory fish, and recorded natural parasite infections in the most abundant species. We analysed the stomach contents of common predatory fish to determine whether, and to what extent, non-native gobies were preyed upon by potential definitive hosts, the essential condition for successful completion of the parasite life cycle. The necessity to investigate parasite fitness when infecting both the non-native and local hosts was pointed out by Kelly *et al*. [28]. They found higher parasite infection in non-native trout compared to native fish in New Zealand, but failed to observe parasite ‘spill-back’. We, therefore, performed a semi-natural study to test parasite fitness in the definitive host. By feeding commercially reared pikeperch (*Sander lucioperca*) with *B. polymorphus* metacercariae recovered from naturally infected hosts (both native and non-native fish species), we investigated the effect of the second intermediate host origin on adult parasite life history traits, such as establishment, survival, maturity and fecundity and their consequences for subsequent parasite dynamics.

## Methods

### Fish sampling

The density of 0+ juvenile fish potentially serving as second intermediate hosts of *B. polymorphus* was surveyed along the shoreline zone (410 m sampled in total) of the lower River Morava (Danube basin, Black Sea drainage) near the town of Lanžhot, Czech Republic, in August 2011. Additional samples were collected in August 2012 and 2013 as a control for goby population dynamics. Fish were sampled by electrofishing using a continual sampling method. Fish density was evaluated as CPUE (Catch per Unit Effort) and expressed as the number of fish per 100 m of shoreline. Individual fish were measured (to the nearest 1 mm) and the majority released back to the river.

Ten 0+ juvenile fish species collected in August 2011 were transported to the laboratory and examined for the presence of metacercariae of *B. polymorphus*. An additional sample of larger round goby individuals was examined in November as a control for fish length potentially affecting metacercariae size. Three fish species (white bream *Abramis bjoerkna* as the most abundant local host, round and tubenose gobies as non-native hosts) were selected for the subsequent experimental study.

Predation of the potential definitive host of *B. polymorphus* on fish intermediate hosts was investigated in the same region. Four fish species were selected, based on their susceptibility to this parasite [18] and their relative abundance in the study area. Wels catfish *Silurus glanis* (Siluridae), European perch *Perca fluviatilis,* pikeperch *Sander lucioperca* and Volga pikeperch *S. volgensis* (Percidae) were collected monthly by electrofishing from January to December 2011. Immediately after capture, fish were anaesthetized with clove oil, killed by cutting the cervical spine and stored in a cool box. In the laboratory, fish standard length and total weight were measured and stomach contents were examined. In addition, the stomach contents of adult gobies (*N* = 49 and 56 for tubenose and round gobies, respectively) were examined for the presence of adult *B. polymorphu*s from May to November 2011 to find out whether gobies also serve as definitive hosts for this parasite [21].

### Metacercariae identification

Cysts containing viable metacercariae of *B. polymorphus* were individually removed from the fish host tissue, placed on a microscope slide and the cyst wall gently disrupted. The released metacercariae were flattened by covering with a cover glass and preserved in 4 % formaldehyde or fixed in 70 % EtOH. Formaldehyde-preserved parasites were stained with acetic carmine for identification based on morphological parameters. A sub-sample of individual metacercariae originating from three intermediate host species selected for experimental study was measured using the Digital Image Analysis package MicroImage 4.0 for Windows (Olympus Optical co., Hamburg, Germany). All measurements (length and width of the metacercariae, primordial ovary, first and second testes), unless specified otherwise, are in μm.

EtOH preserved specimens were subjected to molecular identification based on sequencing of ITS-2 ribosomal DNA fragments. The genomic DNA of 3 *B. polymorphus* metacercariae was isolated by using the QIAamp DNA Mini Kit (Qiagen) according to the manufacturer’s recommendations and stored at −20 °C. The concentration of DNA was measured (NanoDrop 1000, Thermo Scientific). Each 25 μl of PCR reaction contained the following: 12.5 μl 2× concentrated EmeraldAmp GT PCR Master Mix (Takara), 5.5 μl H_2_O, 1 μl of 10 μM forward 5′- GCATCGATGAAGAACGCAGC-3′ and reverse 5′-TCCTCCGCTTATTGATATGC-3′ universal primers designed for ITS-2 region (195–510 bp) according to Yao *et al*. [29] and 25 ng of DNA template (5 μl). Amplification proceeded in a My Cycler (Bio-Rad) following the protocol: 94 °C, 5 min; 35 times 94 °C, 30 s; 50 °C, 30 s; 72 °C, 45 s and a final 10 min extension at 72 °C. Twenty five μl of the PCR products were separated in 1 % agarose during the electrophoresis and purified (MinElute® Gel Extraction Kit, Qiagen). The obtained PCR products were sequenced (3130 × l Genetic Analyzer, Applied Biosystems); sequences were adjusted (DNAStar-Lasergene Core Suite software tool) and compared to the NCBI database (Basic Local Alignment Search Tool - BLAST).

### Experimental study

The experiment was undertaken in semi-natural conditions in outdoor fiberglass tanks (1.35 × 1.35 × 0.9 m) located in the facilities of the Institute of Vertebrate Biology (IVB) ASCR in Brno, Czech Republic. Young-of-the-year (parasite free) pikeperch (*Sander lucioperca*) were used as the experimental definitive host for *B. polymorphus*. Fish were obtained from a commercial hatchery in the Czech Republic where they were kept in an indoor recirculation system and fed with a commercial formulated diet. Fish standard length ranged from 47 – 65 mm with mean ± SD (56.4 ± 3.3 mm). During July 2011, approximately 250 pikeperch were transferred to the IVB, where they were housed and allowed to acclimatize in the outdoor tanks for one week. Prior to the start of the experiment, a sub-sample of 14 fish was dissected to confirm they were parasite-free.

As a source of *B. polymorphus* metacercariae, naturally infected fish intermediate hosts (white bream, tubenose and round gobies) were used. Fish were sampled by electrofishing and transported from the River Morava to the laboratory and kept in aerated aquaria prior to dissection. 0+ juvenile fish were selected to avoid using individuals infected during the previous year. Force-feeding was used to infect naïve pikeperch with metacercariae of *B. polymorphus*. Individual fish were placed in a small dark box (5 × 10 cm) and a piece of muscle tissue was inserted into the fish oral cavity. Fish were observed for 10 min to ensure whether the muscle was swallowed or regurgitated. Fish that regurgitated muscle were removed from the experiment. After infection, fish were transported to the outdoor experimental tanks.

For the experiment, pikeperch were randomly allocated to five groups; fish standard length did not differ among these groups. The first two groups served as a control: one group was without force-feeding manipulation (*N* = 16) and one group was force-fed with muscle tissue free of metacercariae (to control for the effect of the feeding method and to ensure that the muscle had been examined properly, *N* = 18). These fish were force-fed parasite-free muscle tissue of specific intermediate hosts: white bream, tubenose goby and round goby. None of the control fish died during the first three days, the feeding method was thus established as having no negative effect on pikeperch survival. Three pikeperch groups were subsequently experimentally infected with *B. polymorphus* metacercariae. Treatments consisted of 32 fish exposed to metacercariae of each of three intermediate host species (white bream, tubenose goby and round goby; 96 fish in total). Experimental infection of pikeperch was performed by feeding fish muscle tissue containing a specific number of *B. polymorphus* metacercariae. Fish muscle tissue was examined under a binocular microscope and experimental pikeperch was force-fed tissue containing 10 viable metacercariae. This low infection dose corresponded to natural infection of small-sized fish as a potential prey for 0+ juvenile pikeperch. Muscle tissue originated from 20–25 hosts per intermediate host species to increase the chance that the results would reflect the typical characteristics of the parasite population [30].

**Fig. 1:**
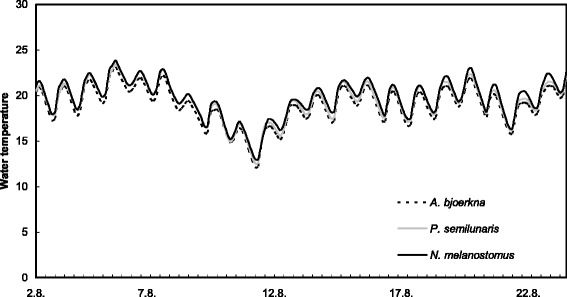
Water temperature (°C) in experimental tanks over the course of the experiment

After infection the fish were transported to six outdoor tanks, each containing 16 pikeperch. Each tank (with both experimental and control fish) was furnished with artificial plants as shelters. Fish condition was monitored visually twice each day and the presence of dead fish was recorded. Dead fish were immediately removed from the tank. Fish were fed daily *ad libitum* with the same formulated pellet food they received in the hatchery from which they were obtained, supplemented with frozen chironomid larvae. Stress was minimized by locating tanks in natural light and temperature conditions and with minimum disturbance. Water temperature was measured eight times daily using data-loggers (HOBO Water Temp Pro v2), with water temperature ranging from 11.5 °C during the night to 24.6 °C during the day (Fig. 1). Mean water temperatures were comparable among the tanks and treatment groups.

Experimental parasite development occurred over 21 days. Based on the results of a preliminary study focused on fluke development in the definitive host, half (8 spec.) of the pikeperch were randomly selected from each tank 8 DPI, killed with an overdose of clove oil and dissected for the presence of flukes in their intestines. The number of flukes was recorded and individual parasites were preserved according to standard methods (see above). After 21 DPI the remainder of the fish were removed from the tanks, euthanized and dissected, with adult and juvenile parasites processed to analyze their maturity and fecundity. At the end of the experiment control fish were also dissected and checked for the presence of *B. polymorphus* flukes. Ethical approval: The maintenance and care of all animals in the experiment complied with legal requirements in the Czech Republic.

### Parasite maturity and fecundity

Adult flukes recovered from the host 21 DPI were placed in individual wells in a multi-well plate, each containing 1 ml of salt solution and allowed to release eggs at room temperature. After ca. 30 min of incubation, the number of released mature eggs and presence of immature, damaged or small eggs was recorded. The number of released eggs was classified into 4 categories: 0 – no eggs, 1 – 1–20 eggs, 2 – 21–50 eggs and 3 – 51 and more eggs. Each fluke was then preserved and mounted for further analysis.

Parasite maturity was evaluated at both 8 and 21 DPI. The number of flukes (1) without eggs; (2) with a predominance of immature eggs; (3) with a comparable proportion of immature and mature eggs; and (4) with a predominance of mature eggs, was recorded. Dark eggs were considered as mature, transparent eggs as immature. The number of eggs was calculated for a sub-sample of 20–22 adult specimens per all intermediate host species (21 DPI). A total number of mature or mature and immature eggs in the uterus was determined and used as an indirect measure of parasite fecundity [31]. The number of uterine eggs at a particular time was used as an instantaneous measure of parasite fecundity reflecting possible differences between the intermediate host species [32]. Length and width of the fluke was measured using Digital Image Analysis (see above). In addition, the number of damaged (deformed) and small (half normal size) eggs was calculated.

### Data analysis

Parasite abundance (mean number of parasites per all fish examined), intensity of infection (range in number of parasites in infected hosts) and prevalence (proportion of infected hosts) were assessed for the three intermediate host species used in the experiment (white bream, round and tubenose gobies). Mean abundance of *B. polymorphus* metacercariae in the fish intermediate host was compared using a Kruskal-Wallis test followed by a Multiple Comparison Test. The same method was used for a comparison of the number of released mature eggs among the flukes originating from the three intermediate hosts. The difference in survival among groups was tested using Survival Analysis (multiple-sample test). The number of living flukes found in the intestine of the definitive host was compared among treatment groups with Analysis of Variance (ANOVA). Differences between the number of flukes 8 and 21 DPI were tested with a *t*-test. GLZ (Generalized linear models) with a binomial distribution were used to analyze differences in the number of flukes with mature/immature eggs and differences in the frequency of damaged and small eggs released. The association between intensity of egg release by adult flukes 21 DPI and total number of flukes infecting the definitive host, as well as the association between the number of eggs and fluke length were examined with a Spearman rank correlation test. Due to the significant relationship between numbers of eggs and fluke size, Analysis of Covariance (ANCOVA) was used to compare number of eggs among flukes originating from the three intermediate host species with fluke length as a covariate. All tests were performed using Statistica 10 for Windows (StatSoft, Inc., 2010).

## Results

### Fish intermediate host and metacercariae

Overall mean CPUE (number of fish per 100 m) of 0+ juvenile fish collected in the littoral section of the River Morava was 51.5, with white bream, chub (*Squalius cephalus*) and tubenose goby being the three most abundant species (Table 1). The proportion of gobies in the littoral fish community tended to increase from 2011 to 2013, with an overall stable fish density (Table 2). Of the 13 species collected in 2011, 10 species were examined for presence of bucephalid metacercariae. The metacercariae were morphologically identified as *B. polymorphus*. The DNA fragment (593 bp) (GenBank database accession number KR817813) exhibited a 100 % identity with ITS-2 *Bucephalus polymorphus* (GenBank database accession number JQ346725.1 [33]).

**Table 1:** A list of 0+ juvenile fish species collected in the littoral zone of the River Morava in August 2011, fish standard length (SL) with mean and range, CPUE (number of fish per 100 m) and presence/absence of *B. polymorphus* metacercariae in specific fish hosts

Fish species	Fish SL (in mm) mean (range)	CPUE	Parasite presence
Cyprinidae			
*Rutilus rutilus*	52	0.24	+
*Squalius cephalus*	41.6 (24–58)	10.88	+
*Leuciscus idus*	37.7 (32–41)	1.71	n.i.
*Chondrostoma nasus*	40.8 (37–46)	0.98	+
*Aspius aspius*	44.7 (38–49)	1.71	+
*Alburnus alburnus*	30.8 (19–49)	2.20	+
*Barbus barbus*	36.0 (23–47)	9.51	+
*Abramis bjoerkna*	43.7 (31–57)	16.34	+
*Rhodeus amarus*	42	0.24	n.i.
Percidae			
*Perca fluviatilis*	36	0.49	-
*Sander lucioperca*	66	0.24	n.i.
Gobiidae			
*Proterorhinus semilunaris*	27.5 (20–34)	9.76	+
*Neogobius melanostomus*	27.6 (20–30)	2.68	+

*+metacercariae of* B. polymorphus *present*

*-metacercariae of* B. polymorphus *absent*

*n.i.* not investigated

**Table 2:** Temporal dynamics in CPUE (number of fish per 100 m) and proportion of cyprinid and gobiid fishes in the littoral fish community of the River Morava from 2011 to 2013

	2011	2012	2013
CPUE (N fish / 100 m)			
Cyprinidae	37.8	21.2	22.7
Gobiidae	24.8	22.8	23.2
Proportion in fish community (in %)			
Cyprinidae	73.8	37.0	45.0
Gobiidae	13.4	39.9	46.0

Except for European perch, in which no metacercariae were detected, all species were found to be parasitized by *B. polymorphus* (Table 1). The three species used in the experiment were infected with 100 % prevalence. Parasite abundance differed among species (H_2,30_ = 10.3, *P* = 0.006), with significantly lower estimates for white bream (19.7 parasites per fish) compared to tubenose goby (57.9) (*P* = 0.004; Table 3).

**Table 3:** Fish standard length (in mm, SL) in native (*A. bjoerkna*) and introduced (*P. semilunaris* and *N. melanostomus*) intermediate hosts of *B. polymorphus*, and its prevalence (P), abundance (A) and intensity of infection (I). Two length classes of *N. melanostomus* were used

	SL (mean ± S.D.)	P (in %)	A (mean ± S.D.)	I (min-max)
Intermediate host species (*N* = 10)				
*Abramis bjoerkna*	42 ± 4	100	19.7 ± 14.3	5 − 45
*Proterorhinus semilunaris*	29 ± 2	100	57.9 ± 24.8	29 − 112
*Neogobius melanostomus* (size 1)	34 ± 2	100	38.2 ± 25.4	6 − 78
*Neogobius melanostomus* (size 2)	65 ± 3	100	37.2 ± 26.6	15 − 97

Measurements of metacercariae of *B. polymorphus* differed among host species (Table 4). Metacercariae of comparable size were found in white bream and tubenose goby, but smaller parasites with less-developed reproductive organs were observed in the round goby. A lower mean size of metacercariae recovered from the round goby was also detected in an additional sample of larger host individuals collected in November (Table 4, *N. melanostomus* (size 2)).

**Table 4:** Measurements of larval *B. polymorphus* (metacercariae), recovered from *A. bjoerkna*, *P. semilunaris* and two groups of *N. melanostomus. N. melanostomus* of size 1 represents 0+ juvenile fish collected in August, size 2 group represents adult/subadult fish (1+ and older) collected in November in the River Morava. Length and width of fluke, primordial ovary and both testes were measured

	Metacercariae size (in μm, *N* = 15)				
Intermediate host species:	Length (mean ± S.D.)	Width (mean ± S.D.)	Ovary (mean)	Testes 1 (mean)	Testes 2 (mean)
*Abramis bjoerkna*	1154 ± 250	280 ± 50	55 × 44	82 × 65	79 × 63
*Proterorhinus semilunaris*	1206 ± 238	245 ± 26	62 × 47	93 × 84	91 × 80
*Neogobius melanostomus* (size 1)	747 ± 150	189 ± 34	34 × 32	45 × 39	43 × 38
*Neogobius melanostomus* (size 2)	892 ± 160	186 ± 33	47 × 38	63 × 54	64 × 57

### Definitive host survey

Fish prey was recovered from all four predatory fish species. However, with the exception of Volga pikeperch, the number of predators with fish in their stomachs was relatively low (Table 5). Only one specimen of pikeperch contained fish (an unidentified cyprinid) in its stomach. Both cyprinid and gobiid fish were found in the stomachs of European catfish (species unidentified) and European perch (European bitterling *Rhodeus amarus*, common barb *Barbus barbus*, tubenose and round gobies along with unidentified cyprinid and gobiid fish remains). In the diet of Volga pikeperch, gudgeon *Gobio* sp., gibel carp *Carassius gibelio*, and unidentified cyprinids, percids and gobiids were recorded. Of the 21 predatory specimens with fish in their stomachs, 7 preyed on gobiid fishes, compared to 17 preying on cyprinids (Table 5).

**Table 5:** Results from a stomach content analysis of predatory fish showing species of predatory fish, number of specimens examined (N), their range of standard lengths (SL), the number of predators that contained a prey species (n), and family to which prey items belonged. Note that some predators contained prey belonging to more than one family

Predatory fish			Prey family			
	N	SL (range, in mm)	n	Cyprinidae	Gobiidae	Percidae
*Silurus glanis*	3	61 − 175	2	1	1	0
*Perca fluviatilis*	28	68 − 191	8	6	4	0
*Sander lucioperca*	17	93 − 172	1	1	0	0
*Sander volgensis*	10	150 − 209	10	9	2	1

Examination of round and tubenose gobies as definitive hosts of *B. polymorphus* failed to show adult flukes in their intestines.

### Fluke establishment and survival

The overall mortality of experimentally infected definitive hosts (pikeperch, *S. lucioperca*) over the entire experiment reached 5 %. Pikeperch mortalities occurred equally among treatment groups (χ = 0.44, D.F. = 2, *P* = 0.80). On the first day of the experiment three fish; i.e. one fish fed with muscle tissue from each intermediate host species, died. Mortality also occurred on the second day with one fish fed with muscle from white bream and on the fifth day with one fish fed with muscle from tubenose gobies. None of the control group fish (both fed by metacercariae free muscle tissue and not force-fed) died before the end of the experiment.

**Fig. 2:**
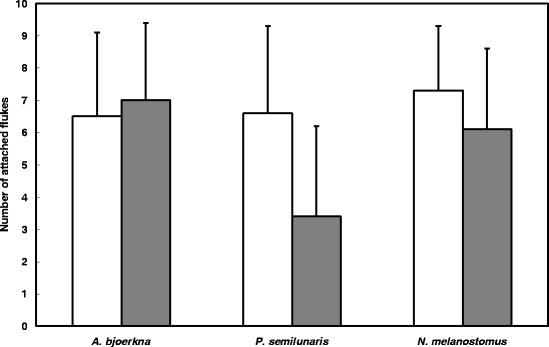
Number (mean ± S.D.) of *B. polymorphus* flukes established in definitive hosts *S. lucioperca* 8 DPI (white bars) and 21 (grey bars) DPI. The x-axis label indicates the original intermediate host species

All flukes were located in the pyloric caeca of experimental pikeperch. No difference in parasite establishment among groups was observed 8 DPI (F_2,41_ = 0.53, *P* = 0.6). A significant decrease in the number of attached flukes from 8 DPI to 21 DPI was observed in parasites originating from tubenose gobies (t_28_ = 3.61, *P* = 0.001). A similar, but non-significant trend in mortality prior to reproduction was found in flukes originating from round gobies (t_29_ = 2.02, *P* = 0.052). The overall survival of flukes originating from tubenose gobies was significantly lower compared to those from round gobies and white bream (F_2,89_ = 5.12, *P* = 0.008) (Fig. 2).

None of the control fish were infected with *B. polymorphus* flukes.

### Maturity and fecundity of *B. polymorphus*

**Fig. 3:**
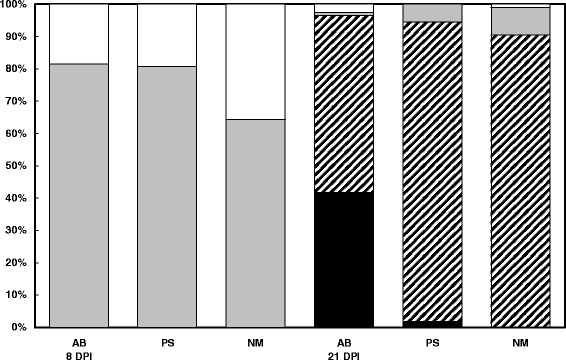
Proportion of flukes recovered from definitive hosts *S. lucioperca* 8 DPI and 21 DPI without eggs (white bars), with only immature eggs (grey bars), with both mature and immature eggs (hatched bars) and with a predominance of mature eggs (black bars). The x-axis label indicates the original intermediate host species: AB = *A. bjoerkna*, PS = *P. semilunaris*, NM = *N. melanostomus*

At 8 DPI none of the flukes were found to contain mature eggs. A significantly higher number of flukes containing immature eggs originated from white bream and tubenose goby compared to round goby intermediate hosts (*N* = 299, Wald = 10.2, *P* = 0.006). Flukes originating from round gobies were more frequently without eggs (Fig. 3). At the end of the experiment 21 DPI flukes of all origins contained mature eggs. The number of flukes with only immature eggs or without eggs was the same for all intermediate host species. The presence of mature and immature eggs was found more frequently in flukes from both goby hosts (*N* = 263, Wald = 38.6, *P* < 0.001). Flukes with a predominance of mature eggs were found more frequently in those originating from white bream compared to tubenose gobies (*N* = 169, Wald = 12.5, *P* < 0.001); no specimens with a predominance of mature eggs were found in flukes originating from round gobies (Fig. 3).

Egg release shortly after removal from the pikeperch intestines was independent of the number of flukes in the fish definitive hosts (*P* > 0.05 for all comparisons) and was observed in 94.3, 97.4 and 98.3 % of parasites from white bream, tubenose and round goby hosts respectively. Flukes originating from white bream released significantly higher numbers of mature and undamaged eggs (H_2,121_ = 24.9, *P* < 0.001) and less frequently damaged or small-sized eggs (Wald = 9.95, *P* = 0.007) compared to both species of goby intermediate host. Size of the released mature eggs did not differ among the intermediate host species origin (Table 6).

**Table 6:** Measurements of adult *B. polymorphus* recovered from juvenile pikeperch definitive hosts experimentally infected with metacercariae from infected intermediate hosts *A. bjoerkna*, *P. semilunaris* and *N. melanostomus*

	Fluke size (in μm, *N* = 20)		Egg size (in μm, *N* = 200)		Number of eggs (*N* = 20)	
Intermediate host origin:	Length (mean ± S.D.)	Width (mean ± S.D.)	Length (mean ± S.D.)	Width (mean ± S.D.)	Undamaged (mean ± S.D.)	Defformed (mean ± S.D.)
*Abramis bjoerkna*	2065 ± 200	368 ± 66	31 ± 2	19 ± 2	674 ± 152	39 ± 31
*Proterorhinus semilunaris*	1986 ± 274	382 ± 70	31 ± 2	20 ± 2	677 ± 197	38 ± 24
*Neogobius melanostomus*	1987 ± 272	362 ± 52	31 ± 2	19 ± 1	534 ± 197	40 ± 31

The total number of undamaged (including both mature and immature) eggs was significantly associated with fluke size (r_s_ = 0.52, 0.59 and 0.52; *P* = 0.016, 0.007 and 0.018 for white bream and tubenose and round goby origin respectively). After correction for fluke length, significantly lower numbers of eggs were found in adult flukes from round gobies (F_2,57_ = 47.5, *P* = 0.016). Numbers of eggs in flukes from white bream and tubenose goby were comparable (Table 6). The total number of damaged (including small and malformed) eggs did not differ among the intermediate host (F_2,57_ = 0.03, *P* = 0.97).

## Discussion

The transmission of native parasites by non-native hosts may lead to ‘spill-back’, when the non-native host is competent for the parasite (e.g. [34, 35]), or to ‘dilution’ effects if the non-native host is susceptible to the parasite, but completion of the parasite life-cycle is disrupted [5, 36]. In this study, we used a combination of field and experimental data to investigate the role of non-native host competence to the local trematode *B. polymorphus* in the population dynamics of this trematode species. We observed high susceptibility to *B. polymorphus* by two non-native goby species (second intermediate hosts), the species showing an increase in density within the littoral fish community in the studied area of the River Morava. Gobies were also found to be heavily preyed upon by piscivorous fish, an essential condition for parasite life cycle completion, indicating that occurrence of non-native hosts in the environment could lead to parasite ‘spill-back’. However, by investigating parasite fitness in both natural and experimental conditions, several mechanisms inhibiting the spread of the parasite in nature were found.

Recent dispersal of *B. polymorphus* in some European waters has been attributed partly to expansion of the first intermediate host, the zebra mussel *Dreissena polymorpha*, and partly to expansion of Ponto-Caspian gobies serving as second intermediate hosts [19, 20]. Although *D. polymorpha* has been present in the River Morava basin for more than 40 years [37], a marked increase in *B. polymorphus* abundance was observed only several years after the introduction of the tubenose goby [38]. Only occasional occurrence of both larval and adult trematodes were reported from the lower Morava basin from the 1950s to the 1990s, with intensity of infection of about 10 worms in definitive hosts and the sporadic presence of metacercariae in the gills of intermediate hosts ([18] and references herein). A dramatic increase in both the prevalence and abundance of *B. polymorphus* metacercariae was found in both native and non-native fish species in this study after goby expansion. Our data showed 100 % prevalence in most of the fish species collected, with a generally high abundance. Based on these field data, non-native gobies are, therefore, assumed to be highly competent hosts for *B. polymorphus* larvae, comparable to local cyprinids. Vulnerability of gobies to *B. polymorphus* larvae appears to be higher than in its native range, as infection parameters were found to be relatively low and with a prevalence of 5 % in the Black Sea basin [39] and up to 35 % in the Sea of Azov basin [21].

Host density is also considered as an important factor positively correlated with the transmission and persistence of parasites [40]. The increasing importance of gobies in the fish littoral community documented in the River Morava (Table 2) places these fish in the role of a potentially important reservoir for this parasite species. Using relatively abundant gobies as intermediate hosts may increase the likelihood of further parasite transmission to piscivorous fish. One third of fish prey recorded in the diet of dominant predatory species of the lower River Morava was represented by gobiids. Moreover, the competence of non-native gobies as definitive hosts could further increase the importance of these species in the population dynamics of *B. polymorphus*. Nevertheless, no adult flukes were observed in the intestine of gobies, possibly because piscivory in the lower River Morava is less frequent than in other regions, probably due to the abundance of other than fish prey [41–43].

The direction and magnitude of parasite occurrence within invaded habitats will depend not only upon the capacity of non-native and native species to serve as competent hosts, but also on the subsequent life history responses of the host and parasite [5, 44, 45]. Metacercariae from non-native round gobies were smaller, with less developed reproductive organs in comparison to parasites from other fish hosts. The size of larval as well as adult parasites may correlate with a range of demographic factors, including host age and number of individuals within a host (e.g. [46, 47]). In comparison to native white bream, round gobies were smaller and infected with higher numbers of parasites, which may have resulted in smaller metacercariae. However, parasites originating from non-native tubenose gobies reached the same size as those from native fish and they showed a higher intensity of infection, despite tubenose gobies being even smaller than round gobies. Further investigation of metacercariae from round gobies showed that they were able to reach the same size as in other species. The mean larval fluke size was, however, still lower, although the parasites were recovered from larger fish collected during late autumn, when the metacercariae were expected to be fully developed. A high proportion of underdeveloped parasites found in the additional sampling of round gobies implied that the development of *B. polymorphus* larvae in round gobies was limited by an immunological [48] or physiological [47] constraint rather than simply due to smaller host size.

Larval size upon reaching the definitive host may positively affect the probability of parasite establishment and initial survival, as well as shorten the maturation time [47, 49]. Transmission of underdeveloped larvae found in round gobies may, thus, have negative consequences on the fitness of adults. However, parasite establishment success measured 8 DPI, as well as the size of adult flukes, was equivalent among the intermediate host species. In addition, survival measured between 8 and 21 DPI was the lowest in parasites from tubenose gobies, a host species with large and well-developed metacercariae. Lower larval size in parasites from round gobies, therefore, probably reflected only a delay in maturity and, consequently, lower numbers of eggs at first reproduction. Compensatory growth after reaching a competent definitive host observed in flukes from round gobies probably resulted in decreased egg production due to increased energy costs for somatic growth. Alternatively, egg production might not have been completed as no flukes with a predominance of mature eggs were found at the termination of the experiment in the group of round goby origin. Due to insufficient data (absence of mature flukes from gobies), the prediction that larger-bodied parasites will tend to have more and larger eggs [31] was thus applicable only at the host intraspecific, but not interspecific level (see Table 3).

As mentioned above, parasites originating from native intermediate hosts started to produce eggs earlier, reached maturity faster and released more mature eggs than those from non-native hosts. Parasites from non-native hosts thus tended to extend the maturation period and delay reproduction. Age at first reproduction shows some plasticity among individuals of the same species. Early maturity allows a parasite to begin producing eggs sooner, whereas delayed maturity potentially allows it to reach a larger size and produce eggs at a higher rate [49]. As the conditions for parasite development in the definitive host were comparable among treatments (water temperature, definitive host species, origin and size), the observed variation probably reflected the factors affecting *B. polymorphus* development in the intermediate host. Taken together, our results showed a reduction in larval growth and lower egg production in flukes recovered from the round goby, lower survival in definitive host in flukes recovered from the tubenose goby, and delayed maturity in flukes originating from both goby species, highlighting the importance of ontogeny in the intermediate host for further parasite development in the definitive host [47].

## Conclusions

In recent years infection by metacercariae of *B. polymorphus* have increased in several water systems in Europe, being attributed to the expansion of both the first intermediate host, dreissenid molluscs, and the second intermediate host species, gobiid fishes, from the Ponto-Caspian region. Our study demonstrated high prevalence and abundance of this parasite species in both native and non-native fish intermediate hosts in the River Morava, occurring after the establishment of gobiid species. The results indicated a role for non-native gobies in population dynamics of *B. polymorphus*, and demonstrated gobies to be competent intermediate hosts for the parasite. However, a semi-natural study showed lower survival and delayed maturity in the definitive host for flukes originating from gobies. Despite considerable ‘spill-back’ of *B. polymorphus* due to higher transmission rate after establishment of non-native gobies, the overall impact is partially buffered by decreased fitness of the *B. polymorphus* developing in gobies.

## Abbreviations

CPUE: Catch per unit effort
DPI: Days post infection
